# Diagnostic Accuracy of Triage Shock Index in Predicting Hyperlactatemia in Patients With Sepsis in the Emergency Department

**DOI:** 10.7759/cureus.90971

**Published:** 2025-08-25

**Authors:** Mervin Christo Chandra Babu, Vimal Koshy Thomas, Anoop T Chakrapani

**Affiliations:** 1 Emergency Medicine, Watford General Hospital, Watford, GBR; 2 Emergency Medicne, Aster Medcity Kochi, Kochi, IND; 3 Emergency Medicine, Kerala Institute of Medical Sciences, Trivandrum, IND

**Keywords:** emergency department, hyperlactatemia, severe sepsis, shock index, triage

## Abstract

Objective: Sepsis is a condition if detected early, can substantially reduce morbidity and mortality. This condition is often diagnosed late, increasing the clinical and logistical challenges in the emergency department. Shock index (SI), defined as the ratio of systolic blood pressure (SBP) to heart rate (HR), has proven advantageous for detecting hemodynamic compromise early. The primary outcome of the study aims to determine the diagnostic accuracy of triage SI in predicting hyperlactatemia (>36 mg/dL) in patients with sepsis presenting to the emergency department. The objective was to determine the diagnostic accuracy of triage SI in predicting hyperlactatemia (lactate ≥36 mg/dL) in adult ED patients diagnosed with sepsis, using ROC analysis.

Methods: This was a prospective cross-sectional study conducted in a tertiary care center in South India from August 2016 to July 2018. Adult patients presenting to the emergency department with suspected infection were screened for sepsis. The triage, vital signs, and basic laboratory tests, including initial serum lactate levels, were measured. Analysis included calculation of sensitivities, specificities, and positive and negative predictive values for the primary outcome.

Results: The analysis of 150 patients in our study revealed good sensitivity (81.6%) and negative predictive value (89.2%) for triage SI in predicting hyperlactatemia in sepsis. However, lower specificity (51.8%) and positive predictive value (36.5%) suggest limitations in precisely confirming these outcomes.

Conclusion: The study concluded that SI greater than 0.68 is a sensitive bedside tool in the early prediction of hyperlactemia in patients with sepsis, and it can be used with other vital signs for effectively triaging patients for giving prompt ED care. Further multi-center studies are required to assess for further validation to incorporate into clinical practice.

## Introduction

Sepsis is a life-threatening condition resulting from a dysregulated host response to infection, leading to organ dysfunction [1]. It remains a major global health burden, particularly in low- and middle-income countries, where delayed recognition and limited resources contribute to high mortality. Early identification of sepsis and timely resuscitation are crucial for improving outcomes, especially in emergency department (ED) settings where rapid triage decisions are essential [2].

Traditionally, vital signs such as heart rate and blood pressure have been used to assess circulatory status [3]. However, these may remain within normal limits during the early stages of sepsis due to physiological compensation, potentially leading to underdiagnosis. Shock index (SI), defined as the ratio of heart rate to systolic blood pressure, is a simple bedside parameter that may help identify patients with early hemodynamic compromise. It has been studied extensively in trauma and obstetric emergencies and is increasingly being explored in septic patients as a non-invasive marker for poor perfusion and adverse outcomes [3,4].

Elevated serum lactate levels are recognized as a surrogate marker of tissue hypoperfusion and are associated with increased morbidity and mortality in sepsis [4]. Hyperlactatemia, often defined as a lactate level ≥4.0 mmol/L (36 mg/dL), is used to guide resuscitation and risk stratification in septic patients. However, lactate measurements are not always immediately available in resource-limited EDs, highlighting the need for accessible triage tools like SI [4,5].

Given the limited data on the utility of SI as a triage tool in sepsis, particularly in the context of predicting hyperlactatemia, further investigation is warranted. This study aimed to evaluate the diagnostic accuracy of triage SI in predicting hyperlactatemia in adult patients presenting with sepsis in the emergency department [3].

In 2017, an estimated 48.9 million cases and 11 million sepsis-related deaths were recorded globally, and 85% of these cases were from low and middle-income countries [2]. Severe sepsis is associated with very high mortality, and early detection with aggressive resuscitation initiated in the ED improves outcome in these patients. Management of sepsis and septic shock is time-dependent, which gives the ED an important role in the care of these patients. Patients with systemic inflammatory response syndrome (SIRS) and a probable or confirmed source of infection are diagnosed to have sepsis. A patient having sepsis with one or more organ dysfunction is diagnosed as severe sepsis [3]. 

Sepsis continues to be a burden and is often unrecognised in the ED. Although blood pressure and heart rate are good indicators of early circulatory compromise, sepsis may go underdiagnosed. The SI is recognized as a more effective marker for assessing the severity of shock and may serve as an early indicator of circulatory compromise in sepsis [4,5].

SI is considered a better marker for assessment of the severity of shock than heart rate and blood pressure alone. SI is a parameter that may serve as an early indicator of circulatory compromise in sepsis. There is a paucity of literature correlating SI in sepsis [4,5]. In critically ill patients, the physiological compensatory mechanisms can maintain blood pressure despite a reduced circulating blood volume. In this context, SI serves as a valuable early warning indicator of shock and may serve as a simple and non-invasive tool for the prompt detection of critically ill patients with sepsis [6].

In patients diagnosed with sepsis, elevated lactate levels are regarded as an objective surrogate marker of tissue hypoperfusion due to accelerated aerobic glycolysis driven by excess beta-adrenergic stimulation. Hyperlactatemia, defined as serum lactate ≥ 4.0 mmol/L (36mg/dL), is a recognised marker of sepsis and has been associated with significant short-term mortality. Studies have also shown that persistent hypotension requiring vasopressors and hyperlactatemia despite volume resuscitation are associated with more than 40% mortality in patients with sepsis [7,8]. Guidelines recommend early measurement of lactate levels in patients with sepsis and resuscitation to be guided to normalise lactate levels [1]. Lactate-guided resuscitation has been associated with a significant reduction in mortality and shorter intensive care unit (ICU) stay [1,9]. In patients with sepsis, SI more than 0.7 is considered a reliable predictor of hyperlactatemia, indicative of sepsis-induced hypoperfusion. Studies have also shown a positive correlation between an elevated shock index and mortality in these patients [10,11].

This study aimed to assess the diagnostic accuracy of triage SI for the early prediction of hyperlactatemia in patients presenting to the ED with diagnosed/suspected sepsis.

## Materials and methods

Study design and setting

This is a prospective, cross-sectional, single-center observational study conducted in the ED of a tertiary care hospital (Kerala Institute of Medical Sciences, Trivandrum) in South India, which receives approximately 40,000 visits annually. The study was conducted over a two-year period, from August 2016 to July 2018, following approval from the Institutional Ethics Committee. Written informed consent was obtained from all participants or their legally authorized representatives. The study was accepted by the hospital's ethical board committee. The study period was from August 2016 to July 2018.

Inclusion criteria

Adult patients aged 18 years or older, patients presented to the ED with suspected or confirmed infection and patients meeting SIRS criteria (two or more of the following: temperature >38°C or <36°C, heart rate >90 bpm, respiratory rate >20/min, or WBC count >12,000/mm³ or <4,000/mm³) were only included [6-8].

Exclusion criteria

Patients were excluded if they were receiving beta-blockers, calcium channel blockers, or metformin; had a known or suspected malignancy; had already undergone prehospital treatment for sepsis (intravenous fluids, vasopressors, or antibiotics); were pregnant; or presented with trauma or haemorrhage.

Patient recruitment and data collection

Eligible patients were screened at the triage counter by the attending emergency physician. Vital signs (heart rate, systolic blood pressure) were recorded prior to any resuscitative intervention. SI was calculated as heart rate divided by systolic blood pressure (HR/SBP). Simultaneously, venous blood was drawn for laboratory analysis, including serum lactate levels and processed using a point-of-care blood gas analyzer.

Lactate measurement was performed by laboratory personnel who were blinded to SI values. The investigators analyzing the outcomes were also blinded to the lactate data at the time of initial triage assessment.

Outcome measures

The primary outcome of the study was to find out the diagnostic accuracy of triage SI for the prediction of hyperlactatemia in sepsis. Outcome analysis in this study was conducted through several statistical techniques. The relationship between SI and lactate levels was explored using correlation analysis to understand their interconnectedness. Additionally, diagnostic performance was assessed using ROC curve analysis, which helped identify the optimal cut-off value for SI in predicting hyperlactatemia. Further evaluation included the calculation of sensitivity, specificity, positive predictive value, and negative predictive value (NPV) to assess SI's effectiveness in detecting hyperlactatemia. These analyses provided a comprehensive understanding of SI's utility in clinical settings, highlighting its potential strengths and limitations as a diagnostic tool.

Selection of participants

Adult patients presenting to the ED with potential signs of sepsis were assessed by the triage doctor and screened using the SIRS criteria. Those who met the SIRS criteria with a suspected/confirmed source of infection were enrolled in the study using convenience sampling after obtaining consent. After selection of patients at triage, vital signs of the patient were recorded, and SI was calculated. Sepsis blood workup, including venous lactate level, was simultaneously sent to the lab from the triage. The study excluded individuals taking medications affecting blood pressure, such as beta blockers and calcium channel blockers. Furthermore, patients on medications like metformin and those with conditions such as malignancy, potentially influencing baseline lactate levels, were also excluded. Additionally, individuals who had received prehospital sepsis treatment, including intravenous fluid resuscitation, vasopressors, and antibiotics, were excluded from the study.

Sample size estimation

A sample size of 150 patients was calculated using the formula by substituting the values 10 of the specificity of SI (0.42) [6], prevalence of hyperlactatemia (0.16) [6,7], and d (variance), with the absolute precision taken as 20%.

N= Z2(1-α/2)Specificity (1-Specificity)

 d2 * prevalence

= 146.2 ~ 150

Interventions

In patients presenting to the ED with suspected/diagnosed sepsis, an initial lactate value of ≥ 36mg/dL (4mmol/L) was considered as hyperlactatemia; simultaneously, their SI was calculated and documented.

Statistical analysis

Data was collected and entered in an MS Excel sheet (Microsoft Corporation, Redmond, USA) and analysed using IBM SPSS Statistics for Windows, Version 26 (Released 2019; IBM Corp., Armonk, New York, United States). All qualitative variables were expressed as frequency and percentage, and quantitative variables were expressed using mean and standard deviation. Pearson correlation was done to find out the relation between SI and hyperlactatemia. Diagnostic test evaluation was done by constructing an ROC curve, sensitivity, specificity, positive predictive value, and NPV to predict hyperlactatemia and mortality by SI. p-value <0.05 is considered to be statistically significant.

## Results

In this study encompassing 150 patients presenting with suspected or confirmed sepsis at a tertiary care center in South India, a comprehensive analysis of demographic and general characteristics revealed that the majority (70%) of participants belonged to the age group of 50 years or older, with a predominant male representation at 62.7%. During the study period, a significant proportion (31.3%) of participants unfortunately succumbed to the illness. Other general characteristics, including vital signs and laboratory data, are given in Table 1.

**Table 1:** Distribution of study subjects based on demographic and general characteristics (n=150) This is the baseline and demographic characteristics of the patients. Continuous data have been expressed as mean±standard deviation while categorical data have been expressed as frequency and percentage. SBP, Systolic Blood Pressure; DBP, Diastolic Blood Pressure; WBC, White Blood Cells.

Variables	Mean ± SD
Age (years)	58.87 ± 15.21
Body Temperature (Fahrenheit)	100.05 ± 1.43
Heart rate (beats per minute)	99.91 ± 17.32
Respiratory rate (per minute)	28.12 ± 7.13
SBP (mmHg)	122.29 ± 26.69
DBP (mmHg)	69.87 ± 11.98
Shock index (HR/SBP)	0.86 ± 0.29
WBC count (per mm^3^)	14955.33 ± 9755.19
Lactate level (mg/dl)	28.79 ± 22.88

Subsequent analysis based on the established cut-off unveiled nuanced insights into the predictive capabilities of SI for both hyperlactatemia and mortality. While the sensitivity and NPV were notably high at 81.6% and 89.2%, respectively, indicating the test's proficiency in ruling out these adverse outcomes. The specificity and positive predictive value were comparatively lower at 51.8% and 36.5%, suggesting that while SI demonstrates effectiveness in excluding hyperlactatemia, its ability to precisely confirm these outcomes may be limited. These findings contribute valuable insights to the understanding and clinical application of SI in the context of sepsis management, informing potential avenues for further research and refinement of predictive tools.

**Figure 1:**
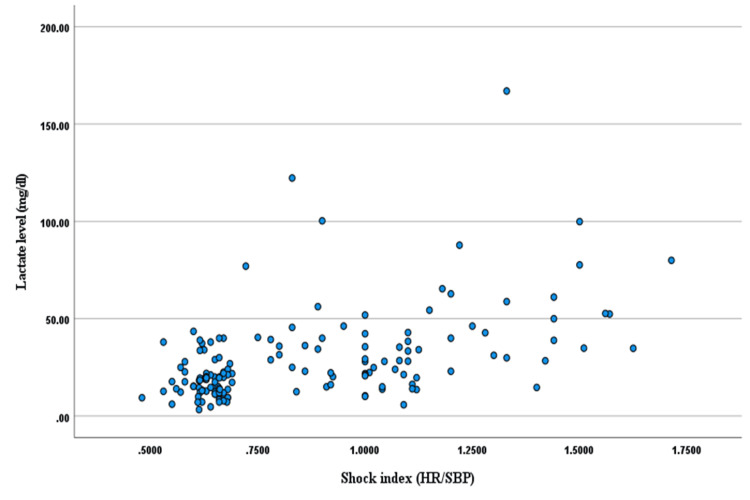
Correlation between the shock index and lactate level among study subjects (n=150) Figure showing the shock index with respect to the lactate level for each case

The study showed the relationship between SI and triage serum lactate levels (Figure 1). The study identified a substantial and statistically significant moderate positive correlation (Pearson correlation coefficient, r=0.50, p<0.001). This finding underscores the interconnectedness of these parameters in the context of sepsis, emphasising the potential utility of SI as a valuable indicator. 

**Figure 2:**
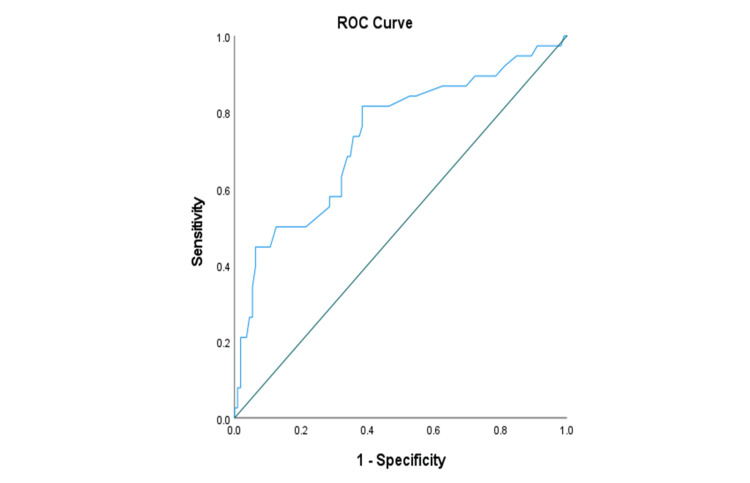
ROC curve to predict the hyperlactatemia outcome by the shock index (n=150) *AUC: 0.735; p-value: <0.001; cut-off score: 0.68

The diagnostic evaluation of SI in predicting hyperlactatemia, assessed through ROC curve analysis, revealed an area under the curve (AUC) of 0.735, indicating a fair predictive test for hyperlactatemia in sepsis patients (p<0.001). The identified optimal cut-off value for SI in predicting hyperlactatemia was determined to be 0.68, providing a valuable threshold for clinical interpretation (Figure 2). 

Table 2 presents the performance metrics of SI in predicting hyperlactatemia among 150 study subjects. The SI demonstrates a sensitivity of 81.6%, indicating a relatively high ability to correctly identify those with hyperlactatemia. However, its specificity is lower at 51.8%, meaning it is less effective in correctly identifying individuals without the condition. The positive predictive value is 36.5%, suggesting that when SI indicates hyperlactatemia, there is a relatively low probability that the individual actually has the condition. Conversely, the NPV is high at 89.2%, highlighting that SI is quite reliable in ruling out hyperlactatemia when the test result is negative.

**Table 2:** Sensitivity, specificity, positive predictive value, and negative predictive value of the shock index (n=150)

	Hyperlactatemia
Sensitivity	81.6%
Specificity	51.8%
Positive predictive value	36.5%
Negative predictive value	89.2%

## Discussion

Recognizing severe sepsis in the ED remains a significant challenge, particularly in resource-constrained environments. Early identification is crucial, as it can substantially decrease both mortality and morbidity in critically ill patients. The ability of commonly used tools like triage vital signs, quick sequential organ failure assessment (qSOFA) score, and early warning score (EWS) for early detection of occult shock may be limited largely due to compensatory cardiovascular responses or the presence of medications such as beta-blockers, which obscure abnormal physiological signs [12-14]. This diagnostic uncertainty often results in delayed clinical intervention, thereby increasing reliance on intensive care resources. These limitations highlight the urgent necessity for more reliable and context-sensitive diagnostic strategies to facilitate the prompt and accurate detection of severe sepsis [12-14].

SI has proven to be a valuable tool for predicting shock and has been used extensively in trauma and maternal haemorrhage in predicting adverse outcomes [15]. In our study, we evaluated the ability of SI to predict hyperlactatemia in sepsis, enabling the initiation of early and active resuscitation for these patients. In a retrospective cohort study assessing the predictive capacity of SI for hyperlactatemia, SI greater than 0.7 performed well in predicting hyperlactatemia with an NPV of 95% and a sensitivity of 83% [10]. These results are comparable to our findings, with an NPV of 89.2% and a sensitivity of 81.6%, indicating that SI may aid in ruling out hyperlactatemia in patients with sepsis. While our findings demonstrated favorable NPV and sensitivity for ruling out hyperlactatemia, it's worth noting that the capacity of SI to effectively exclude hyperlactatemia might be constrained. Additionally, our results align closely with the study mentioned earlier.

SI has also been used to predict the use of vasopressors in early sepsis; SI of >0.8 would predict the need for vasoactive substances within 72 hours of admission [16]. The aforementioned study used trends of SI over a period of time till disposition, which differs from our study, as we have used triage SI only. In this pandemic era of SARS-CoV-2, where medical systems are overwhelmed and are struggling to provide effective care with limited resources, triage SI may help to differentiate, diagnose, and predict those patients who require aggressive resuscitation in the ED, thereby improving their outcomes.

These findings are consistent with the results of Berger et al., who reported that SI >0.7 could reliably identify patients at risk for hyperlactatemia in the ED, with similarly high sensitivity and NPV [9]. Furthermore, Wira et al. (2014) [15] showed that an elevated SI (>0.8) predicted the need for vasopressor support within 72 hours of admission in patients with severe sepsis, highlighting its potential role in early resuscitation planning [16]. These studies support our observation that SI is a pragmatic and inexpensive tool that can augment the clinician’s assessment in the initial triage of septic patients. Compared to other scoring systems such as the qSOFA and the Acute Physiology and Chronic Health Evaluation II (APACHE II), SI is easier to calculate and does not require laboratory data [15]. However, qSOFA includes respiratory rate, altered mentation, and systolic blood pressure, which are stronger predictors of mortality, and APACHE II provides a comprehensive assessment of illness severity [14,15]. While these scores have well-established roles in risk stratification, their utility at the triage level is limited due to time and resource constraints. SI, by contrast, can be obtained immediately and may serve as a rapid screening tool to identify patients who warrant urgent further evaluation with more comprehensive scoring systems [16].

It is important to recognize that elevated lactate in sepsis does not always reflect anaerobic metabolism. While tissue hypoxia remains a primary driver, elevated lactate may also result from aerobic glycolysis induced by β-adrenergic stimulation, hepatic dysfunction, or increased metabolic demand. Therefore, lactate elevation alone should not be interpreted as synonymous with hypoperfusion. This complexity shows the importance of integrating multiple clinical parameters, such as SI, mental status, urine output, and oxygen saturation, when evaluating the severity of sepsis.

Limitations

We have employed only SI to correlate with hyperlactatemia. Newer studies have used modified or age-adjusted SI to predict worsening outcomes. This was a single-center study with a sample size of 150. Multicenter studies may be required to validate SI in sepsis.

This study has several limitations that must be acknowledged. First, although the current consensus recommends the Sepsis-3 definition, we primarily used SIRS criteria for identifying septic patients at triage due to feasibility constraints and local clinical practice. This may limit the generalizability of our findings, particularly in settings that have fully adopted Sepsis-3 criteria with SOFA scoring.

Second, while we attempted to control for known confounders by excluding patients on beta-blockers, calcium channel blockers, metformin, and those with malignancies or prehospital sepsis treatment, other unmeasured variables may have influenced SI or lactate levels. These include conditions such as anemia, hepatic dysfunction, renal impairment, or ongoing medications affecting cardiovascular response, which were not accounted for in the analysis.

Third, this was a prospective study conducted in a tertiary care hospital, which may limit the external validity of the results. Additionally, the modest sample size (n=150) restricts the statistical power and may not capture all relevant clinical variations seen in broader populations. Future multicenter studies with larger cohorts, standardized definitions, and comparative scoring systems are needed to validate the role of SI as a triage tool for sepsis.

## Conclusions

The study has concluded that SI is a useful tool in the early assessment of sepsis, showing a moderate positive correlation with serum lactate levels and fair predictive ability for hyperlactatemia. A cut-off value of 0.68 was identified, with high sensitivity and a high negative predictive value, indicating that SI is effective in ruling out hyperlactatemia. However, its lower specificity and positive predictive value suggest limited accuracy in confirming the presence of hyperlactatemia. Overall, SI shows promise as a non-invasive, early indicator to assist in the management of sepsis, particularly in identifying patients at lower risk for elevated lactate levels.

SI is a reliable, fast, and inexpensive tool to predict hyperlactatemia and mortality in sepsis in the hospital setting. Unfortunately, SI is yet to be popular in EDs, especially in resource-limited settings. Triage is often done using baseline vitals. Often, SI is not calculated. The combination of SI, vital signs, and other warning tools may be required in finding “that” sick septic patient early on and thereby reducing mortality. While the triage SI demonstrates reasonable sensitivity in identifying patients at risk for hyperlactatemia, its low specificity warrants cautious interpretation. SI may serve as a valuable adjunct, but not as a standalone diagnostic tool.
